# Tracking metal pollution from illegal gold mining: a health risk assessment in Edfu, Egypt

**DOI:** 10.1038/s41598-024-84281-8

**Published:** 2025-02-01

**Authors:** Kholoud M. AbdelMaksoud, Wael M. Al-Metwaly, Heba M. R. Hathout

**Affiliations:** 1 Georesources, Natural Resources Department, Faculty of African Postgraduate Studies, Cairo University, Cairo University Street, Giza, Cairo, Egypt; 2 Geography and GIS Department, Faculty of African Postgraduate Studies, Cairo University, Giza, Egypt; 3 Animal resources, Natural Resources Department, Faculty of African Postgraduate Studies, Cairo University, Giza, Egypt

**Keywords:** Wadi Abbadi, Carcinogenic, Toxic metals, Ecological risk, Ecology, Environmental sciences

## Abstract

Over the past decade, there has been an increase in small-scale gold mining in the arid southern region of Egypt. Miners extract ore from the Eastern Desert and transport it to Nile Valley farms, where ample water facilitates the processing. In Edfu, Egypt, the lack of economic opportunities prompted resource-constrained farmers to transform their agricultural lands into gold mines. The study utilized a multifaceted approach that integrated various methodologies, including remote sensing technologies, field surveys, chemical analyses, and statistical methods. The study aimed to assess the concentrations of carcinogenic agents and determine the potential human health risks associated with these agents in soil and fish samples collected within the city boundaries. The study examined correlations between various heavy metals (HMs), such as Ni, Pb, Cd, Cr, Cu, and Hg, in Soilsamples collected in 2020 and 2022. The results revealed direct proportional relationships among specific HMs. The Index of Geoaccumulation (Igeo) and Pollution Load Index (PLI) revealed significantly elevated values in both years, indicating potential environmental degradation. Although no carcinogenic hazards were identified, non-carcinogenic risks related to ingestion were observed for both adults and children exposed to mercury (Hg), copper (Cu), and arsenic (As). Contamination Factor (CF) values were also significantly high. Ecological risks were observed in both Soiland water, as well as in Nile Tilapia samples. Hazard Quotients (HQ) calculated for Nile Tilapia indicated potential risks for both adults and children, particularly associated with elevated arsenic (As) levels. This transformation elicited concerns regarding environmental and health implications, leading us to undertake a thorough investigation.

## Introduction

Egypt is the second largest country in Africa in terms of population, with a population that exceeds 112 million. Egypt’s population, which was 27 million in 1960, has grown significantly, approaching 108 million by 2023^1^. The growth continues unabated, evidenced by a 1.56% increase recorded from 2022 to 2023 (United Nations). Egypt’s history of gold mining extends back to the predynastic period, approximately 4000 BC, preceding the population surge. However, by the fifth century, the prominence of these gold mines declined^2^.

Illegal gold mining in Egypt poses a significant threat to the environment, local communities, and the national economy. The Eastern Desert, a vast and remote area with abundant gold deposits, is the primary focus of this illicit activity, which has significant repercussions. The release of HMs into freshwater ecosystems poses significant threats to both human health and environmental integrity. Toxic substances directly harm living organisms and persist in sediments and water for extended periods, resulting in a lasting legacy of pollution^3–6^. In addition, the implications for human health and aquatic life are significant^3,7^. Researchers often utilize fish as reliable environmental indicators to assess the extent of heavy metal contamination in freshwater systems. The mining industry’s extraction of HMs, such as mercury and arsenic, contributes to environmental toxicity, which raises substantial concerns^8^.

Artisanal and small-scale gold mining (ASGM) in the Nile Valley, particularly in Qena, Edfu, and Aswan, has increased significantly since 2011 despite official prohibitions. The ore, originating from the ancient volcanic rock formations of the Red Sea Hills, is transported to the Nile Valley for processing^9^. The cities located on the eastern bank of the Nile Valley provide advantageous conditions, including ample water resources and low-cost labor for the processing of gold ore from the Red Sea Hills. However, this option carries an increased risk of toxic metal contamination for the local population.

Recent research by Refs.^9,10^ indicates an alarming increase in chromium and cadmium concentrations in Nile River samples, exceeding established safe drinking water standards. The findings indicate that mining activities and agricultural practices may significantly contribute to the degradation of the river’s water quality.

Recent investigations in the Eastern Desert of Egypt have examined the negative impacts of gold mining. Reference^11^ performed a thorough evaluation of the health risks linked to illegal mining activities in the Shalten region. Concurrently^12^, conducted a detailed examination of the environmental impacts associated with gold extraction at the Fawkhair mine. This study employed multivariate statistical and chemical analyses to differentiate between natural (geogenic) and human-induced (anthropogenic) sources of contamination, allowing for the identification of illegal mining’s effect on the overall pollution burden.

This study aims to enhance understanding regarding global environmental risk assessment by accurately quantifying HM concentrations in sediment, differentiating between geogenic and anthropogenic sources of contamination through multivariate statistical analyses, delineating the spatial distribution and extent of contamination, assessing ecological and human health risks utilizing ecological risk indices and health risk assessment models. This research offers valuable insights by highlighting the alarming presence of carcinogenic toxic metals in both soil and fish linked to illegal gold mining, thereby indicating a potential environmental crisis.

## Methodology

This study integrates a diverse array of data sources, including field observations, chemical analyses, statistical methods, and remote sensing (satellite imagery) to thoroughly investigate the study area.

### Study area

**Fig. 1:**
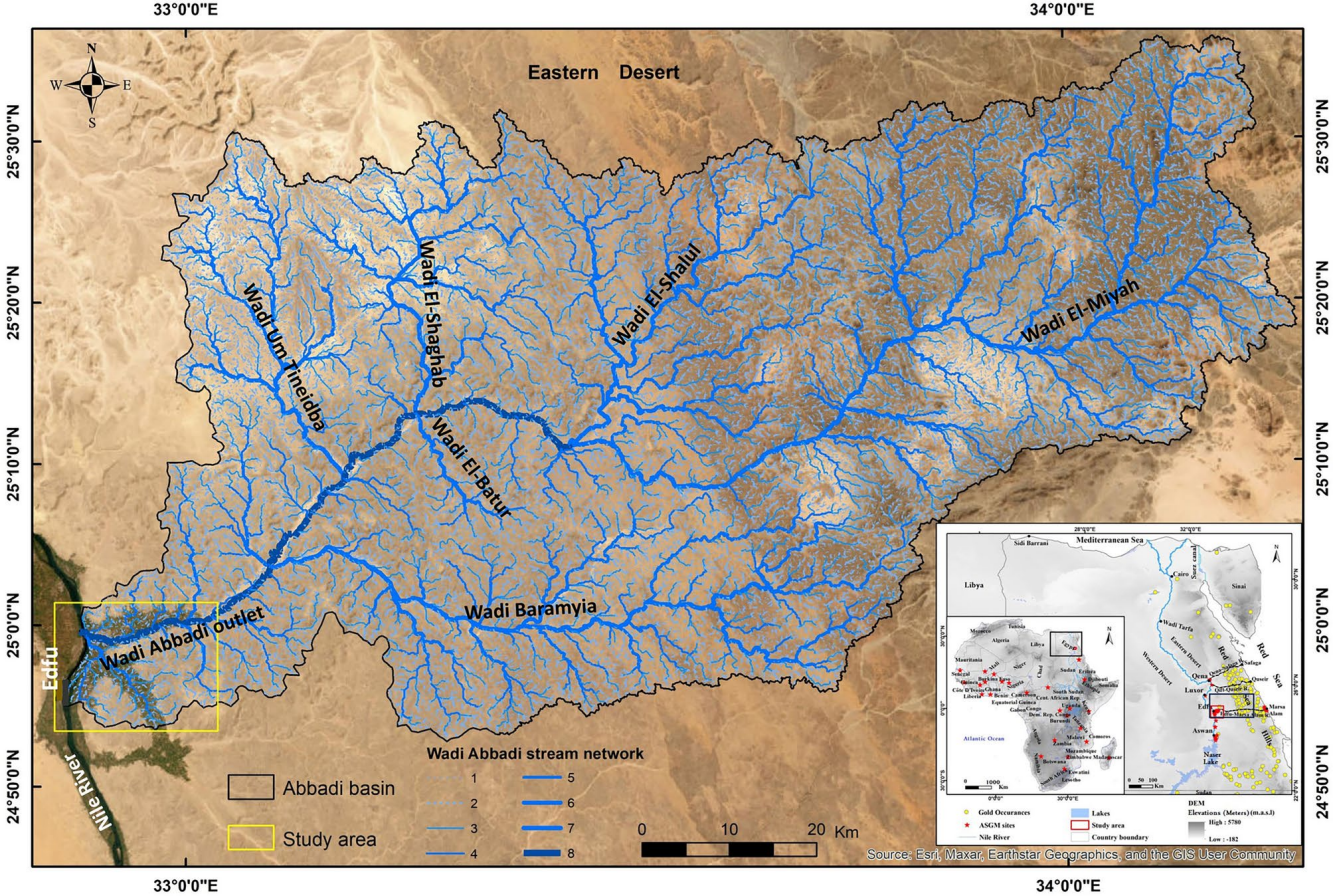
Illustrates the study area (Wadi Abbadi) Edfu city. Source : Esri, Maxer,Earthstar Geographics and GIS ser community.

Edfu City, located in the Nile Valley, serves as the westernmost boundary of the study area. This 10 km segment extends eastward, starting at the intersection of Wadi Abbadi, an east–west valley, with the Nile River. In this study, the easternmost point where Wadi Abbadi meets the Nile will be referred to as the "Wadi Abbadi outlet" (Fig. 1). The 6757 km^2^ basin exhibits a complex geological history characterized by ancient Precambrian rocks from the Neoproterozoic era, which are situated beneath younger formations, including the Upper Cretaceous Taref, Quseir, Duwi, and Dakhla, succeeded by Paleocene Tarawan and Esna deposits, and ultimately capped by Pliocene and Quaternary sediments^13,14^.

A limestone plateau extends southwest of the Wadi Abbadi outlet, running parallel to the Wadi Abbadi. This area contains a substantial network of ASGM sites, exceeding 100 and consistently expanding. Located south of the Wadi Abbadi, these operations significantly alter the landscape. Satellite images confirm that the Abbadi irrigation canal serves as a crucial water source for farmers cultivating fertile lands east of the Nile. The Abbadi drainage canal effectively removes excess water, returning it to the Nile, maintaining balanced irrigation, and promoting healthy crop growth^13^.

### Sample collection

**Fig. 2:**
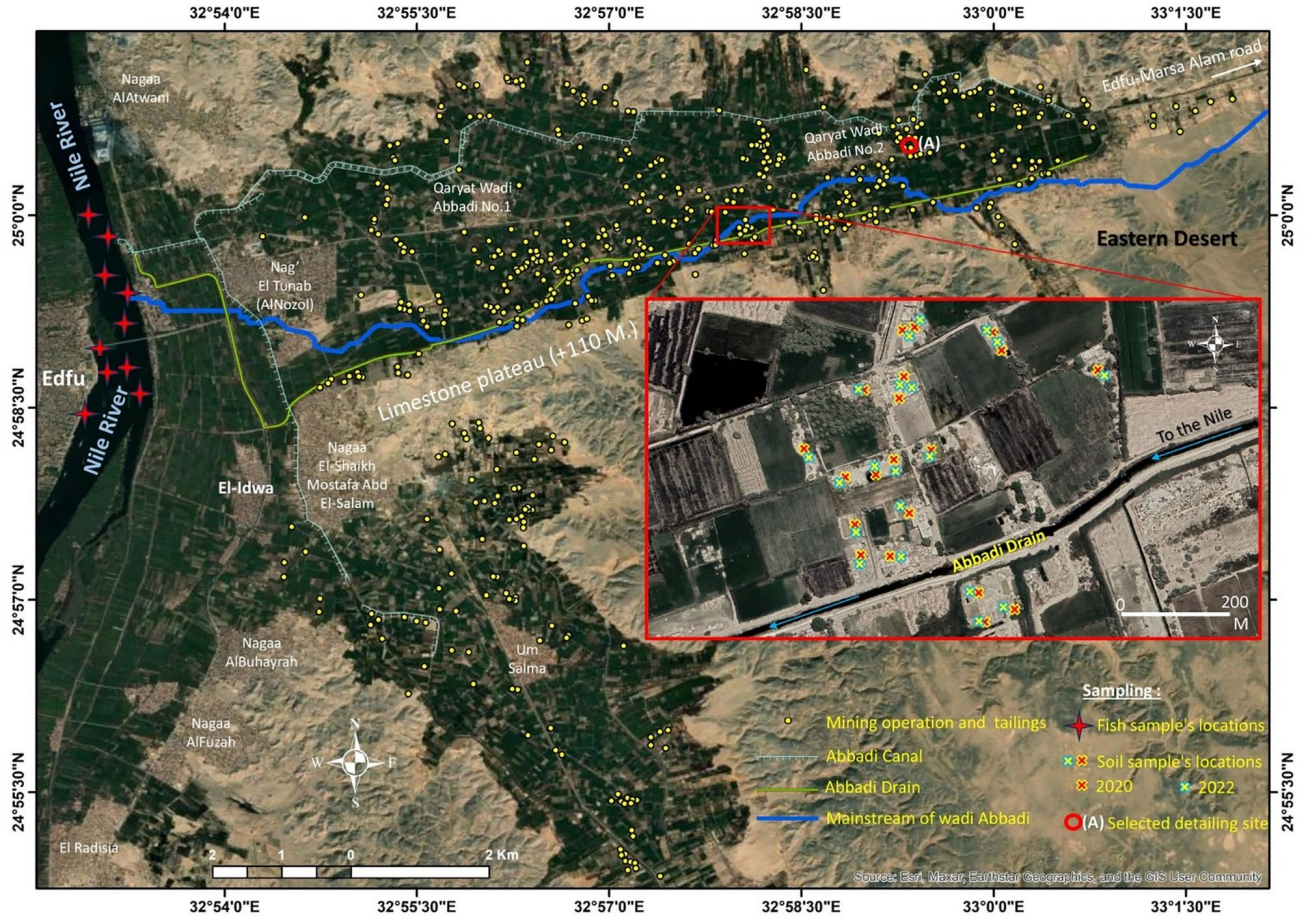
Sample location (soil samples and fish samples). Source: The sites of illegal mining is located according visual interpretation from Google Earth pro 2023 platform then converted from .kmz to points GIS shapefile format, and base map from ESRI, Maxar, Earth star geographics, and the GIS user community.

A systematic sampling protocol was implemented to collect 40 Soil samples, designated Id22-Id23, from a depth of 0–30 cm. Concurrently, 20 fish were obtained from the adjacent Nile River (Fig. 2). Fieldwork was conducted in August under favorable environmental conditions (sunny, calm, and dry) to reduce the likelihood of metal leaching and washing. The region was acknowledged for its significant mining operations. Soil sampling utilized a stainless-steel soil probe, which was thoroughly cleaned between each sampling event to avoid cross-contamination. Five sub-samples were randomly collected within a 1-m radius at each sampling location to ensure sample representativeness. The sub-samples were homogenized and combined to create a composite sample with a total weight of 500 g. Geographical coordinates for each sampling point were accurately recorded with a Garmin eTrex 30 portable GPS device. All collected samples were meticulously labeled and sealed in self-sealing polyethylene bags to prevent contamination and mitigate adverse environmental effects. The samples were subsequently transported to the laboratory for analysis.

A two-year monitoring study (2020–2022) collected 40 soil samples to assess changes in toxicity levels. In contrast to soil samples, which were collected over a two-year period, fish (Oreochromis niloticus) were exclusively collected in 2022. Supplementary Tables S1 and S2 display all samples’ coordinate data and chemical analysis results.

Cairo University approved all experimental protocols (CU-IF-66-24). Additionally, the study procedures complied with the ARRIVE guidelines. We followed the steps outlined in the ARRIVE guidelines to minimize animal suffering and ensure appropriate care and welfare for the experimental animals. We have provided detailed descriptions of the methods and procedures of the animal experiments as well as relevant statistical analyses and data.

The collected samples are placed in small containers, preserved on crushed ice for euthanasia, and then transported to the laboratory, where they are stored frozen at − 20 °C until ready for dissection, tissue processing and digestion.

### Remote sensing

**Fig. 3:**
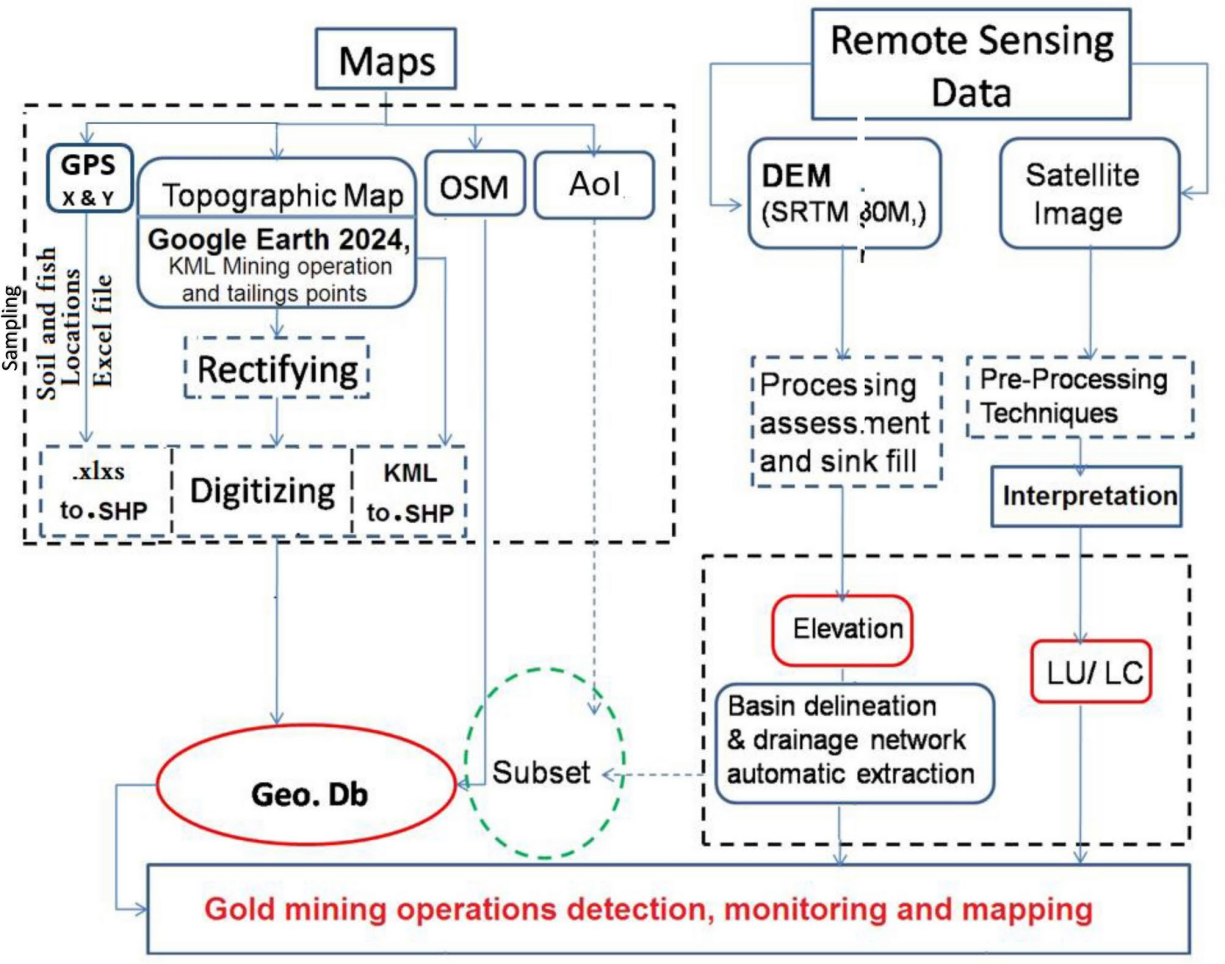
GIS methodology chart.

Detection and monitoring of illegal gold mining activity was achieved through the synergistic integration of remotely sensed data and geographic information systems (GIS) techniques, the methodological design presented in Fig. 3:

- Five topographic map sheets covering the area of interest (AoI) were obtained, namely (Edfu, Al-Kalh, Wadi Al-Shaghb, Wadi um Salama, Jabal Al-Mizan, and Salwa Bahri). These maps were geographically rectified and projected to the Geographic-World Geodetic System (WGS 1984), followed by conversion to the Universal Transverse Mercator (UTM - WGS 1984) metric projection system, which is optimal for calculating the geometric dimensions of the phenomena.
- An extract by mask tool was carried out in the ARC toolbox, a module of ArcGIS, to remove the unimportant appendages along the borders of each map. The Mosaic process was implemented to prepare five map sheets for the digitization of phenomena within the Geo-Db feature classes (Point, Line, and Polygon) designated for the study area. Additionally, using Google Earth 2024 and Wikimapia website, the main names in the study area were identified.
- The digital elevation model (DEM) underwent processing that started with the conversion of its geographical projection system to the metric system (UTM-WGS 1984, Zone 36 North). Subsequently, a Fill process was applied to obtain corrected elevation data for the area. This data served as input for the hydrological analysis model, facilitating the delineation of the Wadi Abadi basin watershed and its stream drainage network. This information was crucial for accurately identifying the study area, its characteristics, and the factors affecting the environmental impacts of illegal gold mining.
- The preparation and processing of the 2020 and 2022 satellite images for the Area of Interest (AoI) were conducted and visually interpreted. The illegal gold mining areas identified using Google Earth 2024 were saved in a KML/KMZ file, subsequently converted to a .SHP file, and then transformed into a feature class-point file stored in the Geo-Db of the studied area, utilizing the metric projection system (UTM-WGS 1984, Zone 36 North).
- The GPS Sampling locations points for soil and fish were exported to Excel file format and then converted from (. Xlxs) extension to a (.SHP) file and converted to the feature class-point file saved in the studied area Geo-Db with metric projection system (UTM-WGS 1984, Zone 36 North).

### Chemical analyses

Digestion of sediment: The Soilsamples were maintained at low temperatures during transport and then frozen at − 20°C until testing for HMs. Prior to testing, the samples were equilibrated to room temperature, placed in designated clean beakers, and dried at 50°C until moisture loss was achieved. The dried sediments, now resembling fine dust, were further ground and passed through a 2 mm mesh plastic sieve to ensure uniformity. In the ratio of 1:3, which is one part hydrochloric acid, to three parts nitric acid, they are digested in a potent concentration of acids known as aqua regia. Then, 20 ml of the freshly prepared mixture of aqua regia was added to a 2 gm sample and boiled gently in a water bath until it shrunk significantly. The digested sample was filtered using a Whatman 0.42 μm filter paper into a 50 mL volumetric flask and topped to the mark with distilled-deionized water. The filtrate was analyzed for HMs using ICP-OES.

Digestion of fish samples: The frozen Nile tilapia samples were gently thawed overnight in a room-temperature environment. A plastic knife delicately glided across the thawed fish to avoid any metallic interference, separating the skin from the muscle. The precious muscle tissue was then nestled into pre-acid washed and oven-dried crucibles, ready for their next analytical adventure. Once oven-dried at 50°C to consistent weight, the samples were cooled in a moisture-free desiccator. A 2-g portion of fish muscle was accurately weighed and subsequently digested in triplicate within a clean beaker. Each sample was treated with 18 mL of concentrated nitric acid and heated at 100°C under a fume hood until the telltale brown fumes disappeared. Subsequently, hydrogen peroxide was added for accuracy. Finally, the digested samples were filtered, carefully transferred to volumetric flasks, and diluted with pure water to a final volume of 50 ml. The samples were then stored in pre-acid-cleaned plastic bottles for further analysis. The analysis was conducted at the Department of Food Toxicology and Contaminants, Egyptian National Research Centre. The metal ion concentrations were analyzed using Inductively Coupled Plasma Optical Emission Spectrometer (ICP-OES) Model Agilent 5100 Synchronous vertical dual view (SVDV), Serial No. MY15180008. A blank solution was prepared using distilled water to provide a baseline for comparison. Detection limits of As, Ni, Pb, Cd, Cr, Cu, and Hg were 0.001, 0.0066, 0.0077, 0.002, 0.007, 0.025, 0.002 ppm, respectively. Blanks were analyzed for each set of five samples, and three samples were replicated to determine the recovery rate. Recoveries ranged from 87 to 110%. The relative standard deviations (RSD) for duplicate samples were < 10%.

Toxic Metal Assay: Highly concentrated reference solutions (1000 mg/L) for seven key elements (As, Ni, Pb, Cd, Cr, Cu, and Hg) were crafted using analytical-grade metal salts dissolved in nitric acid. These “stock solutions” served as the foundation for creating weaker “working standards” through dilutions with distilled water. The instrument’s response to varying element concentrations was meticulously assessed and calibrated using these working standards with progressively higher known concentrations. The actual concentration of each toxic metal in Soiland fish samples in mg/kg was calculated using a specific formula:

$$Actual\: concentration \left(\frac{\text{mg}}{{\text{Kg}}^{-1}}\right)=\frac{\text{Digested concentration }\left(\text{mg}.{\text{L}}^{-1}\right)\times \text{volume digested }\left(\text{L}\right)}{\text{Weight of dried sample }\left(\text{Kg}\right)}.$$

### Statistical analysis

Descriptive statistics were utilized to assess the range, mean, and standard deviations of toxic metals in both soil and fish samples. A study area location map was created using ArcGIS software. Statistical analysis was conducted using IBM SPSS 25 software.

#### Data analysis and calculation method

Soil risk assessment.

#### PLI (pollution load index)

The pollution load index (PLI) is a quantitative metric for assessing heavy metal contamination in sediments, utilizing the contamination factor (CF) concept. The CF indicates the concentration of a particular metal in comparison to its natural prevalence in uncontaminated sediments. It is calculated by dividing the measured metal concentration in a sample by its corresponding background value Eq. (1):

1 $$CF=\frac{C sample}{C Background}.$$

PLI values serve as an intuitive indicator of heavy metal enrichment. Values exceeding 1 indicate that measured metal concentrations surpass their pristine levels (Table 1), suggesting anthropogenic impact (Eq. 2)

**Table 1:** Pollution load index (PLI) standard classes.

Index type	Value	Environmental risk grade	References
PLI	zero	Perfection	Maanan et al. 2015
	1	Baseline	
	< 1	Increasing contamination	

2 $$\text{PLI }= \left(CF1\times CF2\times CF3\times \dots .CFn\right)\frac{1}{n}.$$

#### Geo accumulation (I_geo_)

Soil pollution assessment was conducted using the geoaccumulation index (Igeo) as proposed by Müller^15^ Table 2. The Igeo value was calculated according to (Eq. 3)

**Table 2:** The geoaccumulation index standard classes.

Grade	Value	Soil quality
0	Igeo $\le$ 0	Practically uncontaminated
1	0 < Igeo < 1	Uncontaminated to moderately contaminated
2	1 < Igeo < 2	Moderately contaminated
3	2 < Igeo < 3	Moderately to heavily contaminated
4	3 < Igeo < 4	Heavily contaminated
5	4 < Igeo < 5	heavily to extremely contaminated
6	Igeo > 5	Extremely contaminated

3 $$Igeo={\text{log}}_{2}(Cn/1.5Bn).$$

Bn denotes the corresponding geochemical background value present in the native soil, while Cn signifies the measured concentration of each heavy metal found in the mine tailings. A correction factor of 1.5 is typically employed to account for the influence of natural geological factors and potential anthropogenic impacts on background levels, a correction factor of 1.5 is typically employed, as Ref.^16^ suggested. This factor helps characterize diverse sedimentary features, rock types, and potential human alterations. Subsequently, the calculated Igeo value is categorized into one of seven classes, each reflecting a distinct level of HM contamination, as outlined in Table 1.

#### Ecological risk index in soil and fish

The potential ecological risk coefficient (*E*^*I*^_*r*_) of a single element and the potential ecological risk index (RI) of the multielement were computed using the following equations:

4 $${C}_{f}^{i}={C}_{s}^{i}/{C}_{n}^{i},$$

5 $${E}_{r}^{i}={T}_{r}^{i}\times {C}_{f}^{i},$$

6 $$RI=\sum_{i=1}^{n}{E}_{r}^{i},$$

where ${C}_{f}^{i}$ is the pollution coefficient of a single element of "i "; ${C}_{s}^{i}$ is the measured level (sedimentary/water/fish) of HM; ${C}_{n}^{i}$ is the background level of HM.

${T}_{r}^{i}$ represents toxic response factor for As, Ni, Pb, Cd, Cr, Cu, and Hg, with values of 10, 6, 5, 30, 2, 5, and 40, respectively.

#### Calculation of risk value of heavy metals in soil

Following the U.S. EPA’s human exposure risk assessment method, our study evaluates soil exposure risk for residents in Edfu Square. Overall, all seven HMs exhibit chronic non-carcinogenic risks, with Cd, Cr, and Ni also posing carcinogenic risks. This study posits that residents in Edfu Square are primarily exposed to HMs in Soil is hand-mouth contact, inhalation, and skin contact. The non-carcinogenic exposure risk is typically determined by aggregating the risks linked to various elements across three exposure pathways. This approach fails to account for the interactions between different metals and the human body, along with the variability in toxicity. However, this approach does not consider the interactions between various metals and the human body, as well as the variations in toxicity among pollutants^17^. Due to the limited research on fundamental parameters of Soilemission characteristics, this study utilizes the evaluation guidelines for a local site environment and the criteria outlined by the U.S. EPA for soil health assessment. These guidelines are applied to investigate parameters such as uptake rate, particle release, volatile factors, and biological exposure^18–20^. In this study, pollutant exposure is expressed in terms of unit time and unit weight of the human body [mg/(kg·d)]. Formulas (7) to (9) represent the daily average soil dose through hand-mouth feeding, skin contact, and inhalation, respectively. Formula (10) calculates the average daily exposure to carcinogenic HMs through inhalation. Current assessment standards focus exclusively on inhalation, with insufficient reference data regarding carcinogenic exposure from hand-mouth feeding and skin contact. This study exclusively examines the lifelong daily average exposure via inhalation.

The daily average exposure dose through hand-mouth feeding (ADD ing):

7 $$ADD ing=\text{C} \times \frac{\text{Ing R }\times \text{ EF }\times \text{ ED}}{\text{BW }\times \text{ AT}}\times {10}^{-6}.$$

The daily average exposure dose through skin contact (ADD dermal):

8 $$ADD dermal=\text{C} \times \frac{\text{SA }\times \text{ SL}\times \text{ ABS}\times \text{EF }\times \text{ ED}}{\text{BW }\times \text{ AT}}\times {10}^{-6}.$$

The daily average exposure dose through inhalation (ADD inh):

9 $$ADD inh=\text{C} \times \frac{\text{InhR }\times \text{EF }\times \text{ ED}}{\text{PEF}\times \text{BW }\times \text{ AT}}.$$

The lifetime average daily dose (carcinogenic) (LADD) in Soilis calculated using Eqs. (4)–(6)

as follows:

10 $${\text{L ADD }}_{\text{in}g}=\left(\frac{\text{Cs}\times EF\times {10}^{-6}}{AT}\right)\times \left( {\left(\frac{\text{Ing R}\times ED}{BW}\right)}_{child}+{\left(\frac{\text{Ing R}\times ED}{BW}\right)}_{Adult} \right),$$

11 $${\text{L ADD }}_{\text{in}hl}=\left(\frac{\text{Cs}\times EF}{AT\times PEF}\right)\times \left( {\left(\frac{InhR\times ED}{BW}\right)}_{child}+{\left(\frac{InhR\times ED}{BW}\right)}_{Adult} \right),$$

12 $${\text{L ADD }}_{dermal}=\left(\frac{\text{Cs}\times SL\times ABS\times EF\times {10}^{-6} }{AT}\right)\times \left( {\left(\frac{SA\times ED}{BW}\right)}_{child}+{\left(\frac{SA\times ED}{BW}\right)}_{Adult} \right).$$

In the equation, ADDing represents the daily average exposure to soil particles through hand-mouth feeding. ADDinh represents the mean daily exposure to Soilparticles via inhalation. ADDdermal indicates the mean daily exposure to Soilparticles through skin contact. The values of other parameters in the above formulas are referenced from the U.S. EPA soil health risk assessment method^21^, China site environmental assessment guide^18,21,22^, and related domestic and foreign studies^23^. The results are presented in Tables 3, 4 and 5.

**Table 3:** Calculation parameter values of the daily average exposure of heavy metals.

Items	Parameters/unit	Physics meaning	Values	Data sources
Basic parameters	C/mg·kg^−1^	Concentration of heavy metal	95% UCL	This present study
Exposure behavior parameters	EF/d·a^−1^	Exposure frequency	180	Ferreira-Baptista. L *etal.* 2005
	ED/a	Exposure time	6 (child), 24 (adult)	U.S. EPA,2002; Jiang, L.2004; Ferreira-Baptista. L *etal.* 2005
	BW/kg	Weight per capita	15(child), 70(adult)	U.S. EPA,2002; Jiang, L.2004; Ferreira-Baptista. L *etal.* 2005
	AT/d	Mean exposure time	ED × 365 (non carcinogen) 70 × 365 (carcinogen)	U.S. EPA,2002; Jiang, L.2004; Ferreira-Baptista. L *etal.* 2005
Hand-mouth feeding	IngR/mg·d^−1^	Hand-mouth feeding frequency	200 (child), 100 (adult)	U.S. EPA,2002; Jiang, L.2004; Ferreira-Baptista. L *etal.* 2005
Skin contact	ABS/non-dimensional	Skin absorption factor	1 × 10^–3^	U.S. EPA,2002; Jiang, L.2004; Ferreira-Baptista. L *etal.* 2005
	SA/cm^2^	Surface area of skin exposure	1150 (child), 2145 (adult)	Wang, Z. 2008
	SL/mg·cm^-2^.d^-1^	Skin adhesive capacity	0.2 (child), 0.07 (adult)	Ferreira-Baptista. L *etal.* 2005
Inhalation	InhR/m^3^·d^−1^	Respiratory frequency	5.71 (child), 19.02 (adult)	Ferreira-Baptista. L *etal.* 2005
	PEF/m^3^.kg^−1^	Particulate emission factor	1.36 × 10^9^	U.S. EPA,2002; Jiang, L.2004; Ferreira-Baptista. L *etal.* 2005

**Table 4:** Exposure doses of heavy metals in the soil of Edfu square in different ways in 2020.

Element (2020)	ADD dermal		ADD inh		ADD ing	
	Child	Adult	Child	Adult	Child	Adult
Arsenic (As)	0.00084725	0.000790767	1.6242E-08	1.15933E-08	9.09382E-06	1.27215E-06
Nickel (Ni)	8.48043E-05	0.000791507	1.62572E-09	1.16042E-09	9.10233E-07	1.27334E-07
Lead (Pb)	0.000278806	0.002602192	7.50566E-09	5.35743E-09	4.20238E-06	5.87877E-07
Cadmium (Cd)	2.02544E-06	1.89041E-05	3.88282E-11	2.7715E-11	2.17397E-08	3.0412E-09
Chromium (Cr)	8.0137E-05	0.000747945	2.93406E-09	2.09429E-09	1.64277E-06	2.29809E-07
Copper (cu)	0.000166012	0.001549447	3.1825E-09	2.27162E-09	1.78186E-06	2.49267E-07
Mercury (Hg)	5.60959E-05	0.000523562	1.07537E-09	7.67585E-10	6.02096E-07	8.4228E-08

**Table 5:** Exposure doses of heavy metals in the soil of Edfu square in different ways in 2022.

Element (2022)	ADD inh		ADD ing		ADD dermal	
	Child	Adult	Child	Adult	Adult	Child
Arsenic (As)	0.004061589	0.004351703	8.34233E-08	5.95463E-08	4.67083E-05	6.53408E-06
Nickel (Ni)	0.002483014	0.000266037	5.1E-09	3.64031E-09	2.85547E-06	3.99455E-07
Lead (Pb)	0.005921096	0.000634403	1.21617E-08	8.68083E-09	6.80926E-06	9.52556E-07
Cadmium (Cd)	7.06849E-05	7.57339E-06	1.45184E-10	1.0363E-10	8.12877E-08	1.13714E-08
Chromium (Cr)	0.001646301	0.000176389	3.38143E-09	2.41362E-09	1.89325E-06	2.64849E-07
Copper (cu)	0.013524658	0.00144907	2.77791E-08	1.98283E-08	1.55534E-05	2.17578E-06
Mercury (Hg)	0.001049589	0.000112456	2.15581E-09	1.53879E-09	1.20703E-06	1.68853E-07

#### Calculation of risk value of heavy metals in soil

The model used in the study utilizes specific formulas (13)-(15) to quantify the carcinogenic and non-carcinogenic risks associated with heavy metals. The assessment of non-carcinogenic risk is conducted through the reference dose for chronic poisoning. If the dose at the receptor is below the reference value, it is deemed safe; otherwise, it poses a risk. The carcinogenic risk associated with soil exposure is assessed by calculating the average daily exposure throughout an individual’s lifetime.

13 $$HQ=\frac{\text{ADD}}{\text{RfD}},$$

14 $$HI=\sum HQi,$$

15 $$Risk=LADD\times SF.$$

In the formulas, HQ characterizes the non-carcinogenic risk of a single contaminant via a specific pathway, and ADD represents the non-carcinogenic risk resulting from that pathway. The RfD represents the maximum allowable contaminant intake per unit weight and time that does not result in adverse reactions (mg·kg^−1^·d^−1^). HI is the overall non-carcinogenic risk across various pathways, where total HI is the sum of all non-carcinogenic risks. Generally, a risk is considered small or negligible when HQ or HI is < 1; if > 1, there is a non-carcinogenic risk. SF is the slope coefficient, representing the maximum probability of carcinogenic effects from a specific dose of pollutants (mg·kg·d^-1^). The term “Risk” denotes cancer risk, with values ranging from 10^–6^ to 10^–4^ considered not posing a cancer risk. The values of RfD and SF for Soilor fish and the results are displayed in tables (11–14). Data management and statistical analyses in this study were conducted using SPSS 25 and Excel 2016.

## Results

### Soil

#### Heavy metals concentration and spatial analysis

**Fig. 4:**
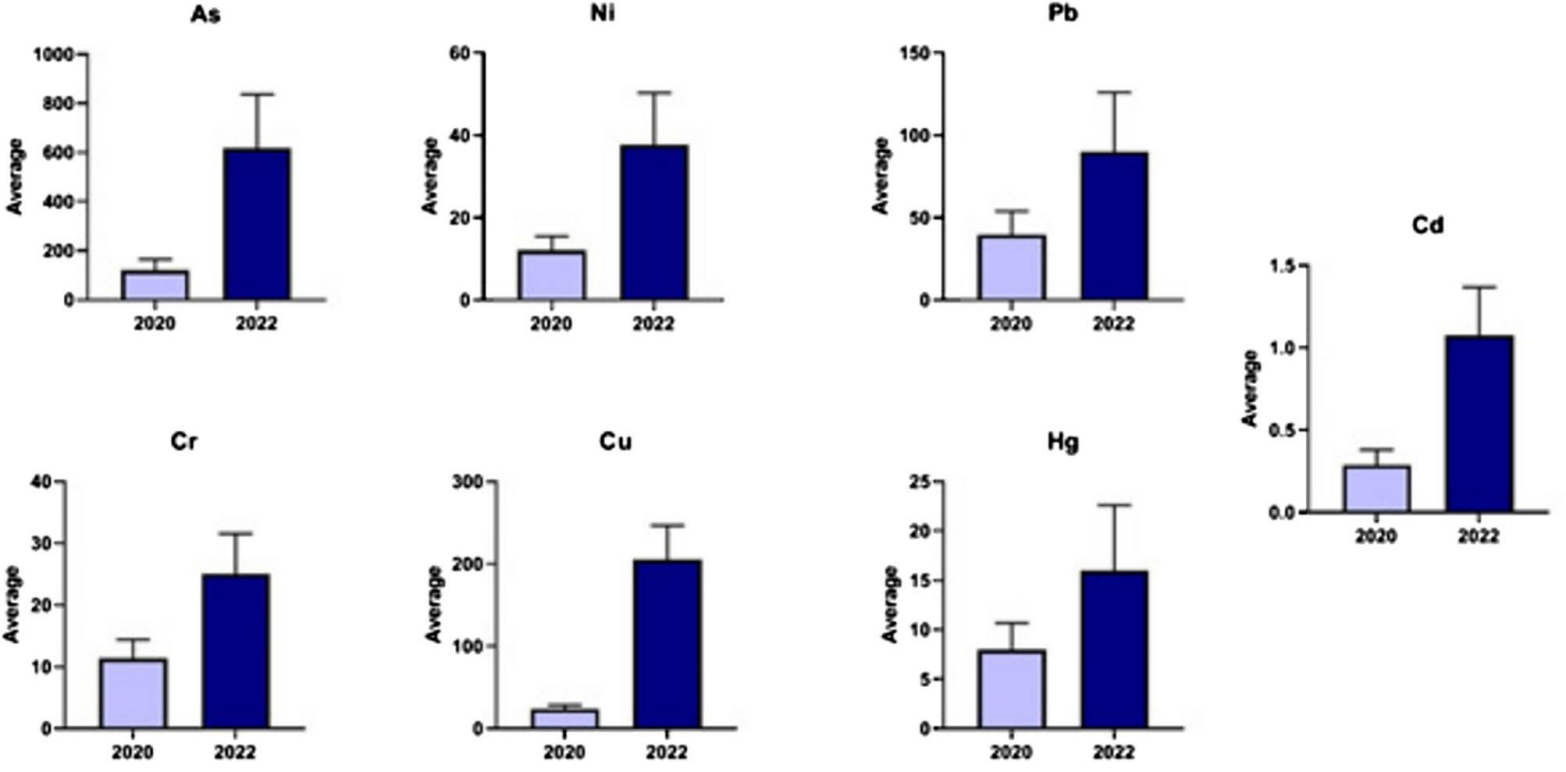

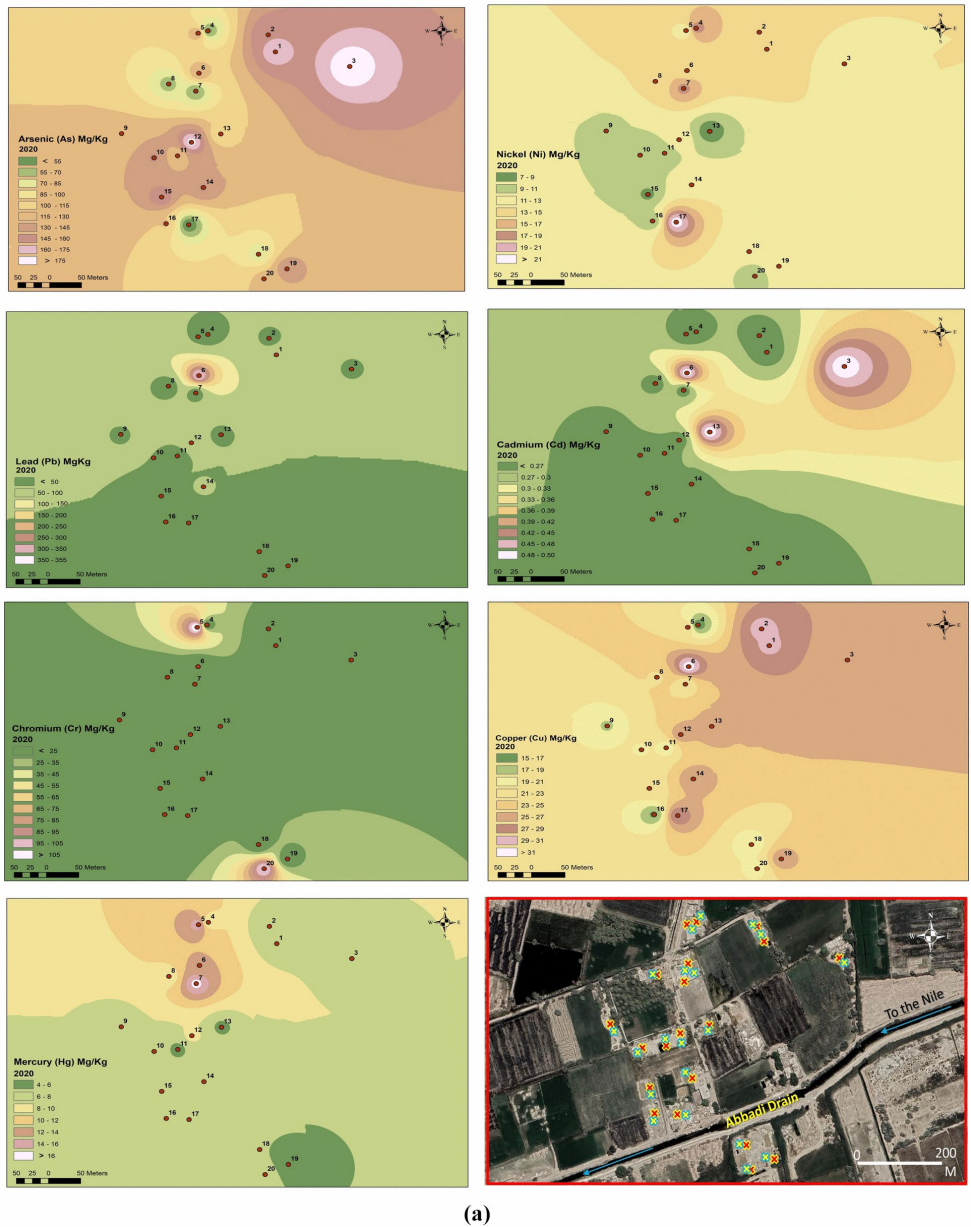

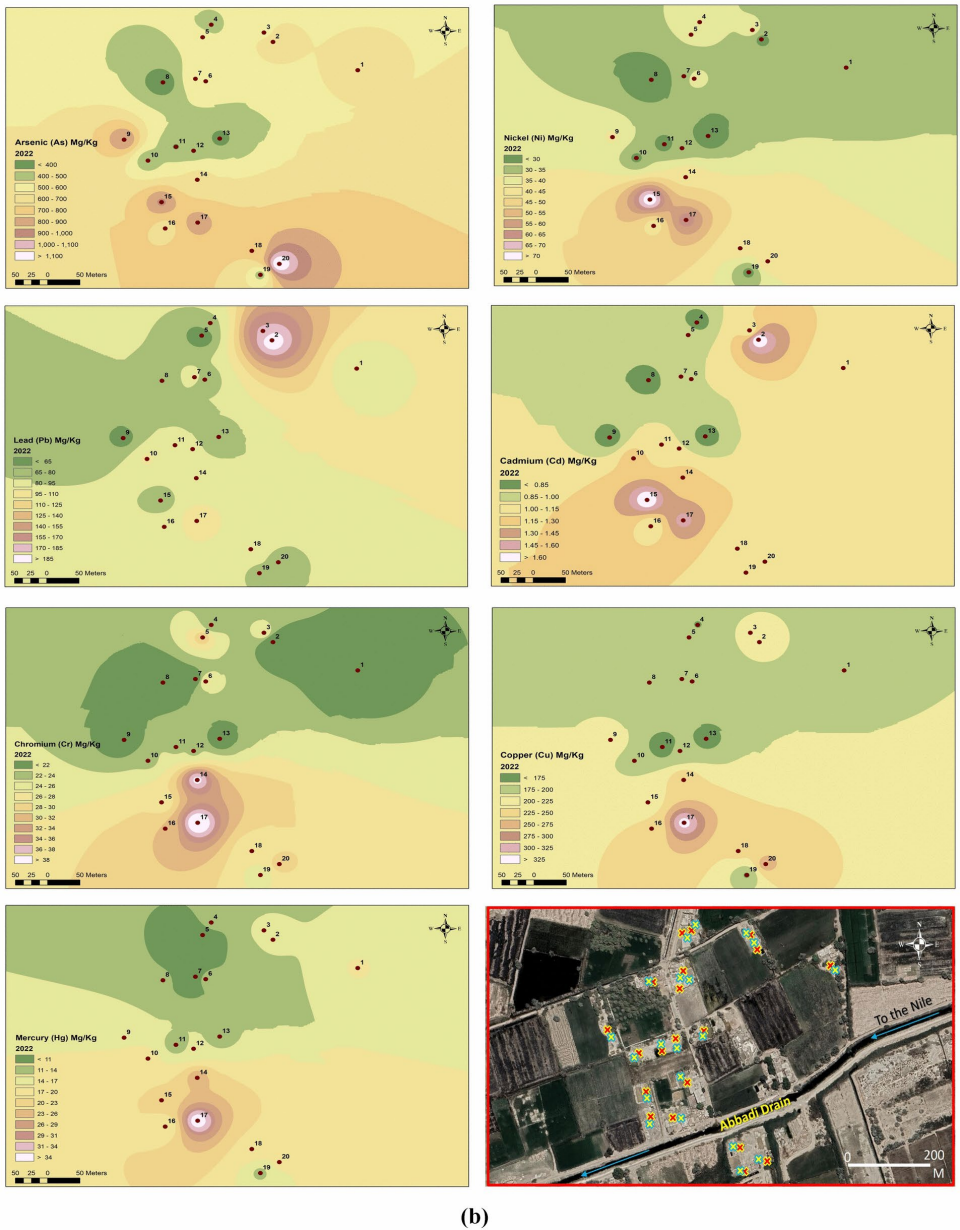
AVERAGE concentration of heavy metals in 2020 and 2022. (**a**) spatial distribution for heavy metals in 2020. (**b**) Spatial distribution for heavy metals in 2022.

This study investigated HM concentrations in soil samples collected in 2020 and 2022. Figure 4 demonstrates a signifcant increase in HM content between the two sampling years. Table 6 summarizes the basic statistics for 2020 samples, including the geoaccumulation index (Igeo) for arsenic (As), nickel (Ni), lead (Pb), cadmium (Cd), chromium (Cr), copper (Cu), and mercury (Hg), which were 6.33, 0.68, 2.399, 4.26, 0.60, 5.71, and 4.73, respectively. Additionally, the pollution load index (PLI) for 2020 was 17.3.

**Table 6:** Basic statistical parameters of the mass ratio of heavy metals in soil of Edfu in 2020 (mg/kg).

Element	Min	Max	Average value	Standard deviation	Coefficient variance CV%	Contamination factor	I_geo_	PLI
As	40.75	187.5	120.26	44.71	37.18	120.26	6.33	17.3
Ni	7.5	22.25	12.0375	3.45	28.66	2.41	0.68	
Pb	17.25	67.5	39.575	14.49	36.61	7.92	2.399	
Cd	0.25	0.5	0.2875	0.092	31.86	28.75	4.26	
Cr	6.75	17.5	11.375	3.041	26.7	2.275	0.60	
Cu	15.25	32.25	23.56	4.74	20.1	78.55	5.71	
Hg	5.5	14.75	7.96	2.72	34.22	39.81	4.73	

Figure 4a demonstrates the spatial distribution and concentrations of specific HMs in the study area for 2020. There is a significant accumulation of As, Ni, Cd, Cr, Cu, and Hg, particularly in the eastern region. Figure 4b depicts the spatial variation of HMs in 2022. The figure highlights a considerable rise in the concentrations of all the tested metals within the same study area.

#### I geo

Table 7 presents the corresponding data for 2022 samples, showcasing elevated Igeo values for all examined metals (8.69), Ni (2.33), Pb (3.59), Cd (6.16), Cr (1.74), Cu (8.84), and Hg (5.73). The PLI value for 2022 further substantiates this enrichment, reaching 58.95.

**Table 7:** Basic statistical parameters of the mass ratio of heavy metals in soil of Edfu in 2022 (mg/kg).

Element	Min	Max	Average value	Standard deviation	Coefficient variance CV%	Contamination factor	I_geo_	PLI
As	313.5	1207.5	617.7	219.61	35.55	617.7	8.69	58.95
Ni	22	74	37.7625	12.56	33.25	7.55	2.33	
Pb	48.75	202	90.05	35.97	39.95	18.01	3.59	
Cd	0.75	1.75	1.075	0.29	27.31	107.5	6.16	
Cr	16.5	41.5	25.038	6.53	26.10	5.0075	1.74	
Cu	152	335	205.69	40.96	19.92	685.6	8.84	
Hg	5	36.5	15.96	6.654	41.69	79.81	5.73	

#### Exposure rate

Table 4 shows the exposure rate of HMs for both adults and children in 2020, indicating that ingestion is the primary route of exposure for both demographics. The most significant metals are As, Pb, and Cu. In 2022, the HM exposure rate was wider for both children and adults. Table 8 shows that As, Ni, Pb, Cr, cu, and Hg are of high values in ingestion, as shown in Table 5.

**Table 8:** Ecological risk index for toxic metals in soil 2022.

Element	${C}_{s}^{i}$	${C}_{n}^{i}$	${C}_{f}^{i}$	${T}_{r}^{i}$	${E}_{r}^{i}$	Risk grade
As	617.7	13	47.52	10	475.15	Extremely strong risk
Ni	37.7625	68	0.56	6	3.33	Slight risk
Pb	90.05	20	4.5	5	22.51	Slight risk
Cd	1.075	0.3	3.58	30	107.5	Strong risk
Cr	25.038	90	0.28	2	0.56	Slight risk
Cu	205.69	45	4.57	5	22.85	Slight risk
Hg	15.96	0.4	39.91	40	1596.25	Extremely strong risk

#### HQ

Table 9 highlights non-carcinogenic exposure risk values (hazard quotients, HQ) for adults exposed to various heavy metals through different pathways in 2020. The HQ_ing_ for arsenic (As) via ingestion is 2.824, surpassing the acceptable threshold, indicating potential health concerns. The trend persists in 2022, as shown in Table 10, where the HQ_ing_ for As ingestion increased to 14.506, indicating a significant increase in non-carcinogenic risk.

**Table 9:** Non-carcinogenic exposure risk values (adult) of heavy metal in different exposure pathways in Edfu sediment in 2020 (Safety parameters according to Hu et al. 2012).

Element (2020)	HQ _ing_	Safety	HQ _inh_	Safety	HQ _derm_	Safety	HI
As	2.824168297	Unsafe	3.86444E-06	safe	0.010342655	Safe	2.834514817
Ni	0.004240215	Safe	5.63309E-08	safe	2.35803E-05	Safe	0.004263852
Pb	0.079658932	Safe	1.64844E-06	safe	0.001119766	Safe	0.080780346
Cd	0.00202544	Safe	2.7715E-08	safe	0.00030412	Safe	0.002329588
Cr	0.026712329	Safe	7.3227E-05	safe	0.003830147	Safe	0.030615703
Cu	0.004150303	Safe	5.67905E-08	safe	2.07723E-05	Safe	0.004171132
Hg	0.01869863	Safe	8.77241E-06	safe	0.004010856	Safe	0.022718259
Total	2.959654147		8.76531E-05		0.019651897		2.979393697

**Table 10:** Non-carcinogenic exposure risk values (adult) of heavy metal in different exposure pathways in Edfu soil in 2022.

Element (2022)	HQ _ing_	Safety	HQ _inh_	Safety	HQ_derm_	Safety	HI
As	14.50567515	Unsafe	1.98488E-05	Safe	0.053122613	Safe	14.55881761
Ni	0.013301859	Safe	1.76714E-07	Safe	7.39731E-05	Safe	0.013376009
Pb	0.181258037	Safe	2.67102E-06	Safe	0.001814393	Safe	0.183075101
Cd	0.007573386	Safe	1.0363E-07	Safe	0.001137144	Safe	0.008710633
Cr	0.058796477	Safe	8.43922E-05	Safe	0.004414146	Safe	0.063295015
Cu	0.036226761	Safe	4.95707E-07	Safe	0.000181315	Safe	0.036408572
Hg	0.037485323	Safe	1.75861E-05	Safe	0.008040602	Safe	0.045543511
Total	14.84031699		0.000125274		0.068784185		14.90922645

Safety parameters according to Hu et al. 2012.

#### ERI

Tables 8 and 11 depict the ecological risk indices (ERIs) of heavy metals in the study area for 2020 and 2022, respectively. The data reveal a concerning trend of increasing ecological risk over the two years. 2020: Arsenic (As) exhibited a "strong ecological risk" with an ERI of 92.51. Mercury (Hg) posed an "extremely high ecological risk" with an ERI of 796.25. Other metals displayed "slight ecological risk," with ERIs ranging from 0.25 to 28.75, while in 2022, The ERI escalated to 475.15, signifying an "extremely high ecological risk." Hg ERI further soared to 1596.25, maintaining its "extremely high ecological risk" status. Cadmium (Cd) ERI rose to 107.5, indicating a transition from “slight” to "strong ecological risk."

**Table 11:** Ecological risk index for toxic metals in soil 2020.

Element	${C}_{s}^{i}$	${C}_{n}^{i}$	${C}_{f}^{i}$	${T}_{r}^{i}$	${E}_{r}^{i}$	Risk grade
As	120.26	13	9.25	10	92.51	Strong risk
Ni	12.038	68	0.18	6	1.06	Slight risk
Pb	39.58	20	1.98	5	9.89	Slight risk
Cd	0.288	0.3	0.96	30	28.75	Slight risk
Cr	11.38	90	0.13	2	0.25	Slight risk
Cu	23.56	45	0.52	5	2.62	Slight risk
Hg	7.96	0.4	19.91	40	796.25	Extremely strong risk

### Nile Tilapia

#### Ecological risk

Tables 12, 13 and 14 present the results of the ecological risk assessment of heavy metals in Nile tilapia. The results presented in Table 9 indicate significant ecological risks linked to specific metals such as Arsenic (As), which presents a significant ecological risk quantified at 3454.71. Nickel (Ni) presents an extreme ecological risk quantified at 787.89. Copper (Cu) exhibits an "extreme ecological risk" quantified at 787.89. Copper (Cu) also presents an "extreme ecological risk" of 824.63.

**Table 12:** Ecological risk index for toxic metals in Nile Tilapia.

Element	${C}_{s}^{i}$	${C}_{n}^{i}$	${C}_{f}^{i}$	${T}_{r}^{i}$	${E}_{r}^{i}$	Risk Grade
As	89.82	0.26	345.47	10	3454.71	Extremely strong risk
Ni	65.66	0.5	131.32	6	787.89	Extremely strong risk
Pb	54.88	1	54.88	5	274.38	Very strong
Cd	0.51	0.2	2.525	30	75.75	Strong
Cr	63.44	0.5	126.88	2	253.76	Very strong
Cu	32.99	0.2	164.93	5	824.63	Extremely strong risk

RI = $\sum_{i=1}^{n}{E}_{r}^{i}$; 5671.1; Extremely ecological risk.

**Table 13:** Human health risk index for adult from consumption of toxic metals in Nile tilapia.

Element	Mean con (mg/kg.dw)	ADD(ing)	RfD	HQ	Safety	SF	CR
As	89.82	8.21234E-05	0.0003	0.273744762	unSafe	1.5	0.000123185
Ni	65.66	6.00297E-05	0.02	0.003001486	Safe	1.7	0.000102051
Pb	54.88	5.01714E-05	0.004	0.012542857	Safe	0.0085	4.26457E-07
Cd	0.51	4.61714E-07	0.001	0.000461714	Safe	0.38	1.75451E-07
Cr	63.44	5.80023E-05	0.003	0.019334095	Safe	0.5	2.90011E-05
Cu	32.99	3.01577E-05	0.04	0.000753943	Safe	–	–

Target hazard quotients = THQ = 0.30983886; Total cancer risk = ΣCR = 0.000254839.

**Table 14:** Human health risk index table for child from consumption of toxic metals in *Nile tilapia*.

Element	Mean con (mg/kg.dw)	ADD (ing)	RfD	HQ	Safety	SF	CR
As	89.82	0.000383243	0.0003	1.277475556	Unsafe	1.5	0.000574864
Ni	65.66	0.000280139	0.02	0.014006933	Safe	1.7	0.000476236
Pb	54.88	0.000234133	0.004	0.058533333	Safe	0.0085	1.99013E-06
Cd	0.51	2.15467E-06	0.001	0.002154667	Safe	0.38	8.18773E-07
Cr	63.44	0.000270677	0.003	0.090225778	Safe	0.5	0.000135339
Cu	32.99	0.000140736	0.04	0.0035184	Safe	–	–

Target hazard quotients = THQ = 1.45; Total cancer risk = ΣCR = 0.001189247.

#### HQ

The hazard quotients (HQ) for adults are presented in Table 13. The arsenic (As) level exceeds acceptable limits, with a recorded value of 0.273744762. However, no carcinogenic risks were identified for any of the analyzed metals in the adult population. Table 14 indicates that the hazard quotient (HQ) for arsenic (As) in children is 1.277475556, which exceeds the safety threshold and is higher than the value recorded for adults. No carcinogenic risk was identified for any of the analyzed metals in the pediatric population.

## Discussion

Field investigations of gold mining sites within the study area identified elevated concentrations of seven HMs (As, Hg, Pb, Cd, Cr, Cu, and Ni) compared to established background levels in the surrounding soil. The current study reveals that the most significant risks in soil and tilapia fish are associated with As, Pb, and Hg, particularly in areas where artisanal and small-scale gold mining (ASGM) utilizes mercury amalgamation for gold extraction from ore. This process entails the amalgamation of crushed ore with mercury, resulting in an amalgam that is later subjected to heat to extract gold while volatilizing the mercury. This common practice in illicit mining accounts for the high mercury (Hg) concentrations found in soil samples from the studied site. The volatilization of mercury during gold extraction results in its release into the surrounding environment, posing potential inhalation and ingestion risks for miners and nearby communities^9,11,12^.

Asare^24^ highlights the elevated concentration of arsenic (As) in specific igneous and sedimentary rock formations. This enrichment is attributed to the incorporation of arsenic-bearing minerals, such as orpiment (As_2_S_3_), realgar (As_4_S_4_), and arsenopyrite (FeAsS), within the rock matrix. This elucidates the elevated concentration of arsenic in the analyzed sample, where the primary lithologies employed for ore extraction are igneous rocks.

Reference^25^ observed lower lead (Pb) concentrations in coexisting plagioclases within igneous rocks, reflected by a Pb in K-feldspar/Pb in Plagioclase ratio ranging from 6.7 to 1.7 ppm. In contrast, the substantial Pb concentrations reported by Ref.^24^ at the investigated mining site suggest potential detrimental impacts on the surrounding soil. Furthermore, lead exposure through ingestion, skin absorption, or inhalation poses detrimental effects on human and animal health due to its interactions with various enzymes within organ cells^26^.

**Fig. 5:**
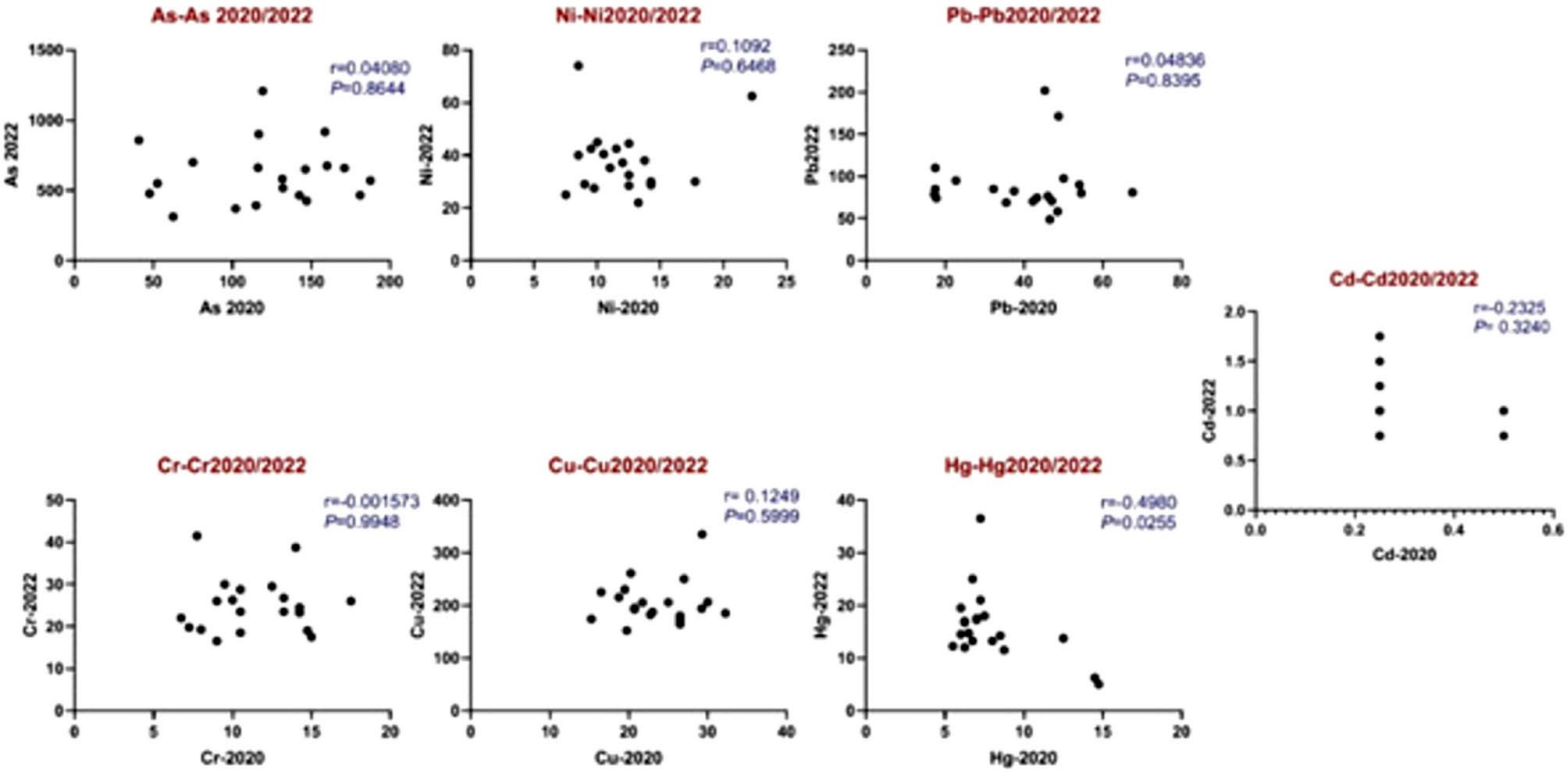
Correlation between metals in both 2020 and 2022.

This study investigates the potential correlation between HM concentrations at a gold mining site in 2020 and 2022 (Fig. 5). The analysis identified proportional relationships among various HMs, such as Ni, Pb, Cr, cu, and Hg. Figure 2 demonstrates a significant increase in gold mining sites, exceeding 130 by 2024. The increase in mining activity may account for the significant rise in the Index of Geoaccumulation (Igeo) and Pollution Load Index (PLI) from 2020 to 2022, indicating possible environmental degradation due to elevated HM release.

**Fig. 6:**
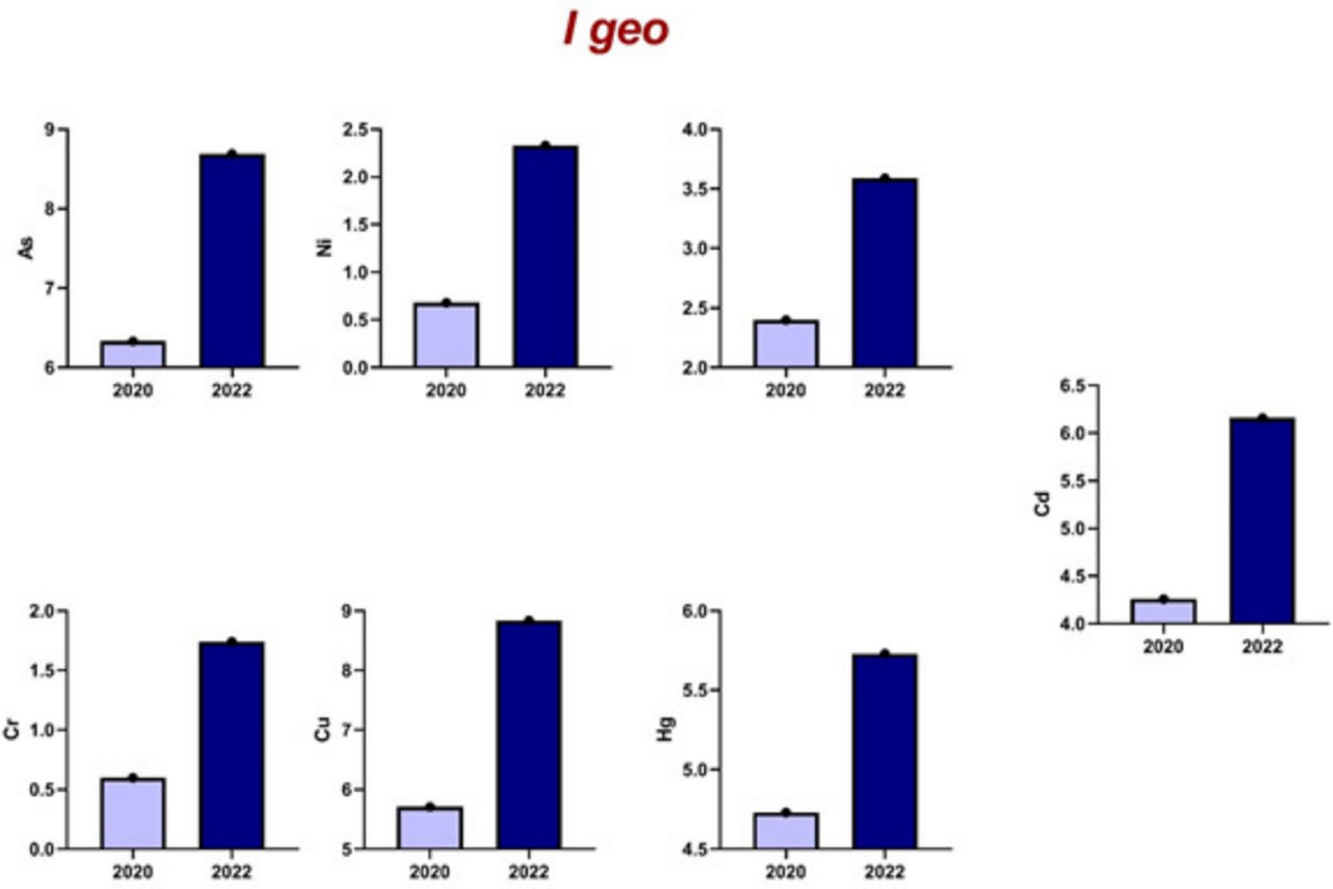
I _geo_ for heavy metals in 2020 and 2022.

**Fig. 7:**
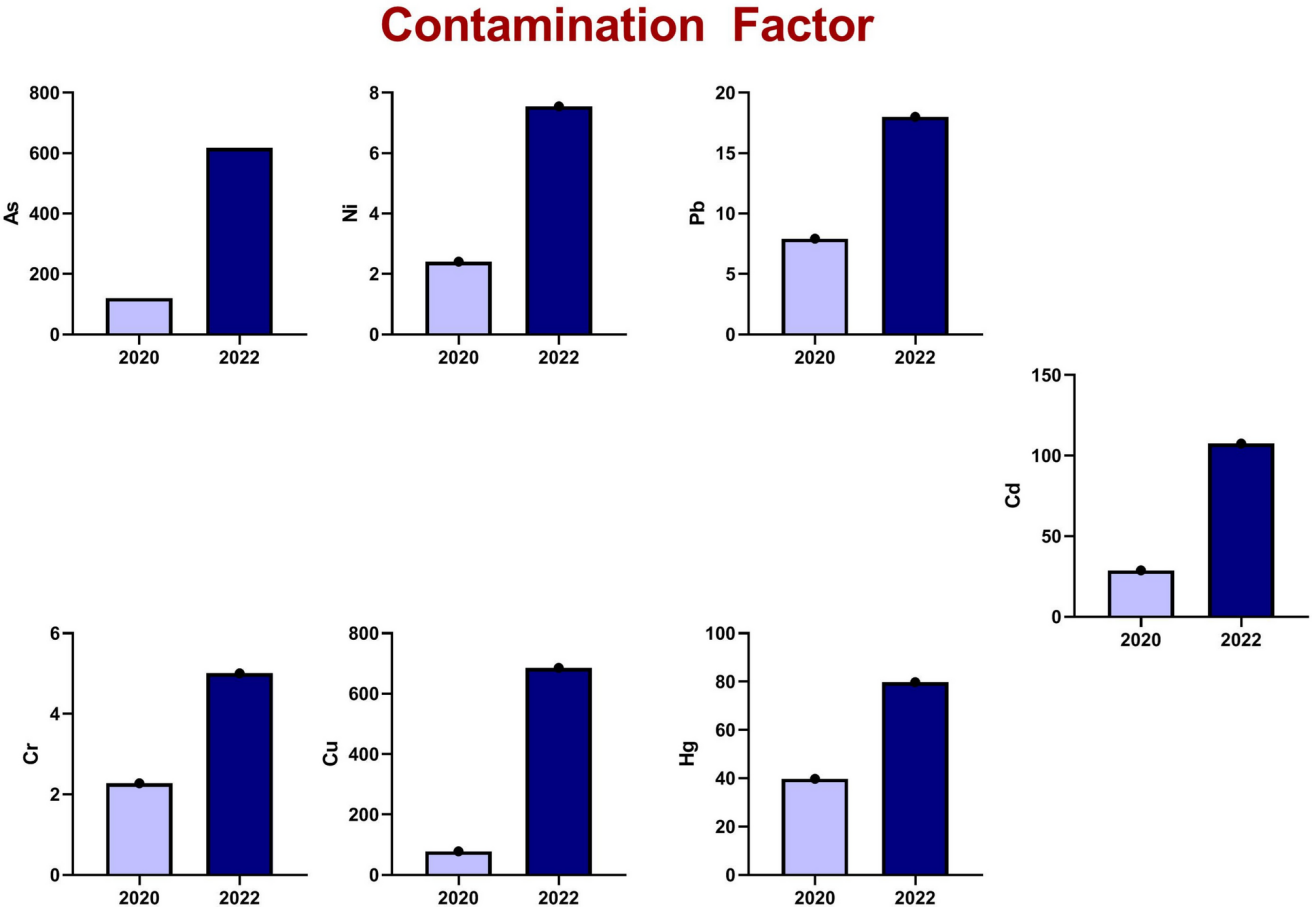
CF for Heavy metals in 2020 and 2022.

Figure 6 shows a significant increase in Index of Geoaccumulation (Igeo) values from 2020 to 2022, transitioning from moderately to heavily and highly contaminated, indicating a pronounced intensification of heavy metal contamination at the studied site. Figure 7 indicates a significant rise in Contamination Factor (CF) values, supporting the trend of increasing HM pollution.

Based on the research of Ref.^11^, which examined the effects of illegal mining on heavy metal concentrations in Egypt’s Eastern Desert, the current study demonstrates a significant rise in the toxicity of the analyzed HMs compared to their results.

**Fig. 8:**
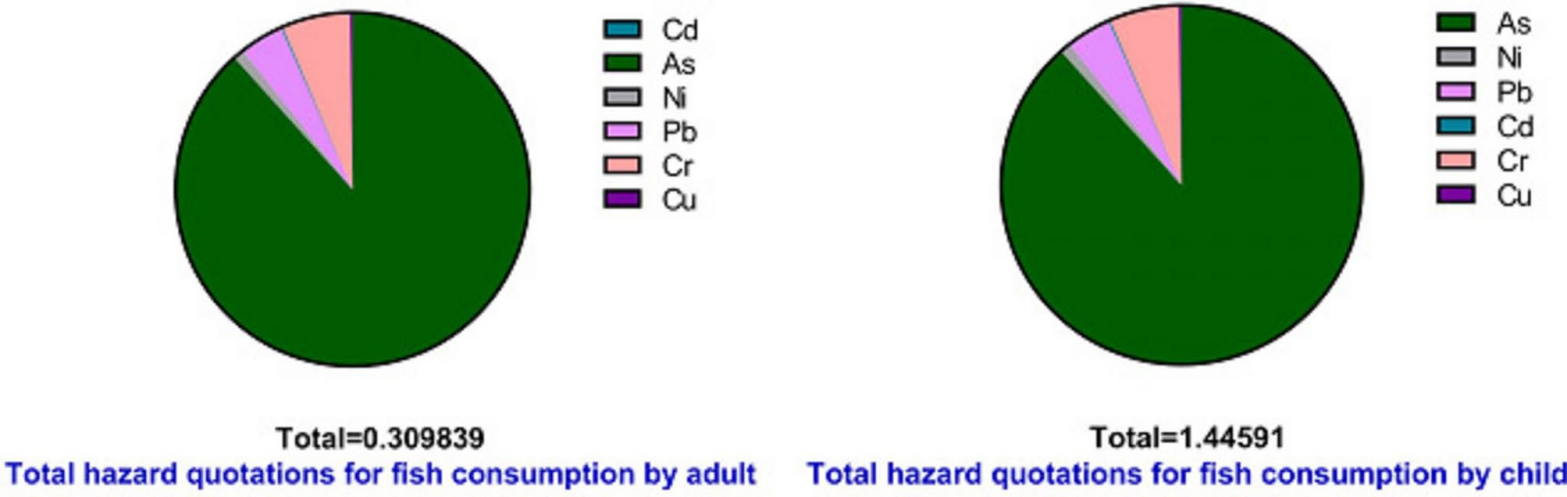
HQ for *Nile tilapia* for both adult and child.

Hazard quotients (HQ) calculated for both adult and child Nile Tilapia indicate potential risks, as depicted in Fig. 8. Notably, children exhibit higher HQ values compared to adults. This finding supports the established principle that children are more vulnerable to environmental hazards due to their lower body weight and higher relative intake rates.

**Fig. 9:**
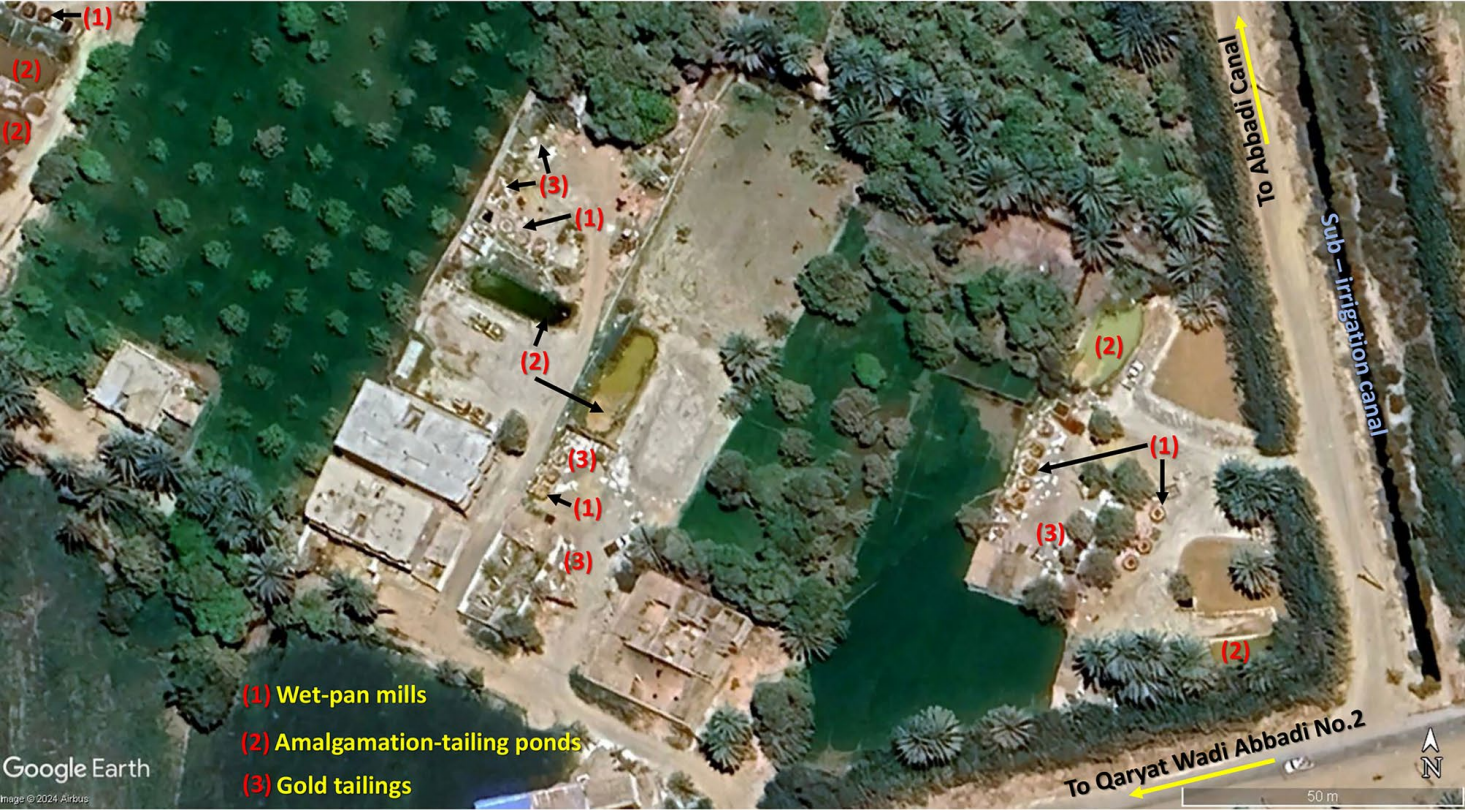
Mining sites illustrating mining operation in each site. Source: Sample points located using (Add x, y) tool and interpolation maps created using IDW spatial interpolation tool, based on Arc-GIS software package.

As shown in Fig. 9, the study area is located near agricultural lands fed by the Nile River. Satellite imagery indicates that illegal gold mining sites are expanding within agricultural lands, resulting in significant environmental consequences for agriculture in the region. Consequently, it is essential to manage the environment surrounding the study area to avert environmental degradation resulting from these illegal activities.

Reference^27^ studied the recent trend in environmental monitoring, which involves integrating geographic data and remotely sensed information within a Geographic Information System (GIS) and spatial distribution framework. The spatial distribution of heavy metals, clearly depicted in Fig. 4a and b, can be used to evaluate the efficacy of mitigation efforts in the region by identifying notable trends in their concentration. This approach facilitates the analysis and monitoring of mining activities, enabling a comprehensive assessment of their environmental impacts. The present study used the same tool to monitor the mining activity in the study area and detect the extendable sites in the last few years.

The findings of this study indicate a significant increase in heavy metal concentrations, highlighting the substantial environmental risks associated with unregulated mining activities. The use of remote sensing techniques to evaluate the spatial distribution of heavy metals and thorough statistical analysis demonstrates the significant impact of mining-related pollution on the surrounding environment, including aquatic ecosystems and human health.

Risk assessment and monitoring are crucial aspects of responsible gold mining. This proactive approach facilitates the identification of existing hazards within the sector, encompassing the degree of environmental degradation and the decline of the health condition in illicit mining districts. Collaboration between the Minerals Commission and health directorates is crucial for an effective risk assessment. This collaboration enhances comprehension of environmental degradation and promotes the incorporation of health and safety risk management strategies within gold processing practices. This comprehensive approach helps to reduce detrimental practices that adversely affect the environment and human health.

The implementation of sustainable mining practices and environmentally friendly technologies is essential for the protection and restoration of rivers and streams. Moreover, promoting active community involvement in decision-making processes is essential for the sustainable preservation of these critical water resources for both present and future generations^28^. The establishment of continuous monitoring programs for fish populations and soil health in the study area affected by illegal gold mining is crucial for effective environmental management. Longitudinal studies can provide essential data regarding the specific impacts on human health, aquatic biodiversity, and soil quality. The scientifically derived data obtained from this research can be utilized to inform evidence-based decision-making processes. This approach can significantly enhance the development and implementation of effective mitigation and restoration strategies.

## Conclusion

Analyzing the spatial distribution of illegal mining sites and pollutant pathways is essential for prioritizing remediation efforts and protecting the health of local communities and ecosystems. Our findings hold the potential to inform critical decisions and shape sustainable solutions for responsible gold mining practices in the Nile Valley. The observed increase in the As, Hg, and Cd ecological risk index suggests a significant deterioration in ecological health between 2020 and 2022. Based on the study results, the following conclusions were made:

- The implemented risk monitoring methodology demonstrated high efficiency over a two-year period. This coincided with the anticipated timeframe for increased vulnerability within the study area. The methodology’s effectiveness facilitated the identification of environments likely to experience heightened pressure in the future.
- Our analysis of Edfu Soilrevealed the presence of HMs such as arsenic (As), mercury (Hg), and lead (Pb). This study identified non-carcinogenic risks from ingestion pathways as the primary concern, while the carcinogenic potential of these specific HMs was deemed negligible, indicating a minimal carcinogenic health risk.
- The elevated ecological risk index associated with arsenic and mercury underscores the necessity for prompt identification and mitigation of their sources to protect the ecosystem. Conversely, other metals present reduced individual risks. Their combined influence must be considered in thorough ecological risk assessments. This observation indicates that unauthorized gold mining activities lead to environmental contamination by hazardous heavy metals, which raises concerns regarding health risks for nearby residents.
- Remote sensing techniques offer unbiased data, corroborating the continuous identification of illegal gold mining activities as key targets of various land-change pressures.
- The primary contribution of this study is its potential to establish a foundation for the development of environmental databases. These databases could be instrumental in monitoring environmental changes and assessing toxicological risks within the Nile River ecosystem, specifically focusing on soil and fish.

Unsustainable mining practices can cause soil erosion, land degradation and loss of fertile land. Chemical pollutants used in gold extraction, such as mercury and cyanide, can contaminate water sources, posing risks to human health and ecosystems. Illegal gold mining results in a loss of tax revenue for the government, which could otherwise be allocated for development and public services. This activity is often associated with criminal organizations and can lead to instability in specific regions.

The Egyptian government has implemented various initiatives to address illegal mining, such as enhanced patrols, more stringent regulations, and public awareness campaigns. Additionally, to reduce the risk associated with such mining. In order to mitigate the risks associated with mining, it is essential to implement stricter laws and regulations to combat illegal gold mining and enhance enforcement efforts. International organizations, including the United Nations Environment Programme (UNEP) and the World Wildlife Fund (WWF), are supporting the efforts of the Egyptian government. The issue is multifaceted and lacks straightforward resolutions. However, collaboration among the government, local communities, and international organizations can facilitate advancements in mitigating the detrimental effects of illegal gold mining.

### Limitation of the study

The illegal nature of the mining operations presented significant challenges to the field study. The stigma associated with the absence of state authorization impeded the acquisition of samples. In addition, the skepticism and distrust that were prevalent in this field posed a challenge to collaboration with researchers.

A further limitation of the study was the lack of suitable facilities, both domestically and internationally, for analyzing cyanide. global ban on cyanide utilization hinders the identification and measurement of this compound in mining regions, where substantial, undisclosed quantities of complex cyanide are utilized for ore extraction. The potential risks associated with cyanide presence in sediments remain uncertain.

## Supplementary Information

Supplementary Tables.

## Data Availability

Data is available from the corresponding author upon a reasonable request.
