# Supplementary material for: Tracking metal pollution from illegal gold mining: a health risk assessment in Edfu, Egypt
Source: Sci Rep. 2025 Feb 1;15:3974. doi: 10.1038/s41598-024-84281-8 (PMC11787331; doi:10.1038/s41598-024-84281-8)
Supplement: Supplementary file 1 — Supplementary Tables. [file 41598_2024_84281_MOESM1_ESM.docx]

S1: Coordinates for Fish samples and chemical analyses:

| **Location ID** | **Coordinates** | | **Results (Mg/Kg)** | | | | | |
| --- | --- | --- | --- | --- | --- | --- | --- | --- |
|  | **x** | **y** | **Arsenic**  **(As)** | **Nickel**  **(Ni)** | **Lead**  **(Pb)** | **Cadmium**  **(Cd)** | **Chromium**  **(Cr)** | **Copper**  **(Cu)** |
| **1** | 32.88532 | 24.99702738 | 0.55 | 0.55 | 5.82 | < 0.05 | 5.81 | 0.27 |
| **2** | 32.88487 | 24.99198921 | 1.35 | 0.677 | 1.35 | < 0.05 | 2.03 | 2.03 |
| **3** | 32.88779 | 24.98963806 | 0.24 | 0.24 | 0.24 | < 0.05 | 0.48 | 0.48 |
| **4** | 32.88409 | 24.98252864 | 2.175 | 0.725 | 1.45 | < 0.05 | 1.45 | 2.9 |
| **5** | 32.88213 | 24.97401973 | 0.236 | 0.473 | 0.473 | < 0.05 | 0.236 | 0.236 |
| **6** | 32.8839 | 24.99947274 | 0.595 | 0.595 | 0.595 | < 0.05 | 1.78 | 1.488 |
| **7** | 32.88708 | 24.98759271 | 0.83 | 0.416 | 0.208 | < 0.05 | 1.66 | 0.83 |
| **8** | 32.88472 | 24.97970427 | 22.12 | 22.12 | 11.06 | < 0.05 | 33.18 | 33.18 |
| **9** | 32.88753 | 24.98051329 | 0.283 | 0.283 | 0.57 | < 0.05 | 0.85 | 0.57 |
| **10** | 32.88862 | 24.97703928 | 0.55 | 0.184 | 0.184 | < 0.05 | 1.28 | 0.74 |

S2: Coordinates and chemical analyses for Soil samples in both 2020 and 2022:

| **ID** | **Coordinates 2020** | | **Coordinates 2022** | | **Results (Mg/Kg)** | | | | | | | | | | | | | |
| --- | --- | --- | --- | --- | --- | --- | --- | --- | --- | --- | --- | --- | --- | --- | --- | --- | --- | --- |
|  |  |  |  |  | **Arsenic**  **(As)** | | **Nickle**  **(Ni)** | | **Lead**  **(Pb)** | | **Cadmium**  **(Cd)** | | **Chromium**  **(Cr)** | | **Copper**  **(Cu)** | | **Mercury**  **(Hg)** | |
|  | **x** | **y** | **x** | **y** | **2020** | **2022** | **2020** | **2022** | **2020** | **2022** | **2020** | **2022** | **2020** | **2022** | **2020** | **2022** | **2020** | **2022** |
| **1** | 32.96812 | 25.00023064 | 32.96925 | 24.99993 | 171 | 659 | 14.25 | 30 | 54.5 | 80 | 0.25 | 1 | 14.75 | 19 | 29.25 | 194.25 | 7 | 17.25 |
| **2** | 32.96801 | 25.00050109 | 32.96807 | 25.00038 | 160 | 675 | 14.25 | 28.75 | 45.25 | 202 | 0.25 | 1.75 | 15 | 17.5 | 30 | 206.5 | 6 | 14.5 |
| **3** | 32.96917 | 24.99999792 | 32.96794 | 25.00053 | 187.5 | 570 | 12 | 37.25 | 48.75 | 171.25 | 0.5 | 1 | 17.5 | 26 | 25 | 205.75 | 6.5 | 14.75 |
| **4** | 32.96716 | 25.00056399 | 32.9672 | 25.00066 | 47.5 | 477.5 | 8.5 | 40 | 17.25 | 78.5 | 0.25 | 0.75 | 6.75 | 22 | 15.25 | 174 | 8 | 13.25 |
| **5** | 32.96702 | 25.00052625 | 32.96708 | 25.00046 | 132 | 516.5 | 11 | 35.25 | 46.5 | 48.75 | 0.25 | 1 | 122.5 | 29.5 | 23 | 187.5 | 15.5 | 6.25 |
| **6** | 32.96703 | 24.99989099 | 32.96713 | 24.99975 | 131.75 | 582 | 13.75 | 38 | 355.5 | 69 | 0.5 | 1 | 13.25 | 26.75 | 32.25 | 185 | 12.5 | 13.75 |
| **7** | 32.96699 | 24.99960796 | 32.96698 | 24.99979 | 52.5 | 550 | 17.75 | 30 | 17.5 | 85 | 0.25 | 1 | 8 | 19.25 | 20.75 | 195 | 16.75 | 5 |
| **8** | 32.96661 | 24.99972117 | 32.96653 | 24.99973 | 62.5 | 313.5 | 13.25 | 22 | 17.75 | 74.25 | 0.25 | 0.75 | 9 | 16.5 | 22.75 | 182.5 | 8.75 | 11.5 |
| **9** | 32.96594 | 24.99893496 | 32.96599 | 24.99882 | 116.75 | 900 | 10.5 | 40.5 | 48.5 | 58.5 | 0.25 | 0.75 | 10.5 | 18.5 | 18.75 | 215 | 6.25 | 17 |
| **10** | 32.9664 | 24.99855129 | 32.96632 | 24.99848 | 147 | 425 | 9 | 29 | 50 | 97.5 | 0.25 | 1.25 | 10.5 | 23.5 | 20.75 | 192.5 | 7.5 | 18 |
| **11** | 32.96673 | 24.99858274 | 32.96671 | 24.9987 | 115 | 392.5 | 9.75 | 27.5 | 37.5 | 82.5 | 0.25 | 1 | 13.25 | 23.5 | 19.75 | 152 | 5.25 | 12 |
| **12** | 32.96693 | 24.99879659 | 32.96696 | 24.99864 | 181 | 465 | 12.5 | 32.5 | 67.5 | 81 | 0.25 | 1 | 14.25 | 23.25 | 26.5 | 180 | 8.5 | 14.25 |
| **13** | 32.96734 | 24.99892867 | 32.96732 | 24.99883 | 102 | 370 | 7.5 | 25 | 42.25 | 70.5 | 0.5 | 0.75 | 7.25 | 19.75 | 26.5 | 164.5 | 5.5 | 12.25 |
| **14** | 32.9671 | 24.99807956 | 32.96701 | 24.99817 | 146.25 | 650 | 12.5 | 44.5 | 54 | 90 | 0.25 | 1.25 | 14 | 38.75 | 27 | 250 | 6.75 | 25 |
| **15** | 32.96651 | 24.99792861 | 32.96651 | 24.99782 | 158.75 | 917.5 | 8.5 | 74 | 47 | 71 | 0.25 | 1.75 | 9 | 26 | 21.75 | 205.5 | 7.25 | 21 |
| **16** | 32.96657 | 24.9975072 | 32.96656 | 24.99739 | 116.25 | 661.5 | 9.5 | 42.5 | 32.25 | 85 | 0.25 | 1 | 9.5 | 30 | 16.5 | 225 | 6.25 | 16.75 |
| **17** | 32.96689 | 24.99748833 | 32.96701 | 24.99749 | 40.75 | 857.5 | 22.25 | 62.5 | 17.5 | 110 | 0.25 | 1.5 | 7.75 | 41.5 | 29.29 | 335 | 7.25 | 36.5 |
| **18** | 32.96788 | 24.9970229 | 32.96777 | 24.99704 | 75 | 700 | 11.5 | 42.5 | 22.75 | 95 | 0.25 | 1 | 10 | 26.25 | 19.5 | 230 | 6 | 19.5 |
| **19** | 32.96828 | 24.99679018 | 32.96789 | 24.99665 | 142.5 | 464 | 12.5 | 28.5 | 46 | 76.5 | 0.25 | 1 | 14.25 | 24.5 | 26.5 | 172.5 | 4.75 | 13.25 |
| **20** | 32.96795 | 24.99663294 | 32.96815 | 24.99683 | 119.25 | 1207.5 | 10 | 45 | 43.25 | 74.75 | 0.25 | 1 | 107.5 | 28.75 | 20.25 | 261.25 | 7 | 17.5 |
